# B Lymphocyte intestinal homing in inflammatory bowel disease

**DOI:** 10.1186/1471-2172-12-71

**Published:** 2011-12-30

**Authors:** Caterina Defendenti, Piercarlo Sarzi-Puttini, Silvia Grosso, Annamaria Croce, Olivia Senesi, Simone Saibeni, Simona Bollani, Piero Luigi Almasio, Savino Bruno, Fabiola Atzeni

**Affiliations:** 1 Laboratory Unit, Fatebenefratelli Hospital, Milan, Italy; 2 Rheumatology Unit, L. Sacco University Hospital, Milan, Italy; 3 Division of Gastroenterology, Fatebenefratelli Hospital, Milan, Italy; 4 Division of Pathology, Fatebenefratelli Hospital, Milan, Italy; 5 GI & Liver Unit, DIBIMIS, Policlinico, University of Palermo, Palermo, Italy; 6 Experimental Medicine, Queen Mary University, London, UK

**Keywords:** Inflammatory bowel disease, inflammation, mucosal immunity, lymphocytes, B1 cells, lymphocyte homing

## Abstract

**Background:**

Inflammatory bowel disease (IBD) is thought to be due to an abnormal interaction between the host immune system and commensal microflora. Within the intestinal immune system, B cells produce physiologically natural antibodies but pathologically atypical anti-neutrophil antibodies (xANCAs) are frequently observed in patients with IBD. The objective is to investigate the localisation of immunoglobulin-producing cells (IPCs) in samples of inflamed intestinal tissue taken from patients with IBD, and their possible relationship with clinical features.

**Methods:**

The IPCs in small intestinal, colonic and rectal biopsy specimens of patients with IBD were analysed by means of immunofluorescence using polyclonal rabbit anti-human Ig and goat anti-human IgM. The B cell phenotype of the IPC-positive samples was assessed using monoclonal antibodies specific for CD79, CD20, CD23, CD21, CD5, λ and κ chains. Statistical correlations were sought between the histological findings and clinical expression.

**Results:**

The study involved 96 patients (64 with ulcerative colitis and 32 with Crohn's disease). Two different patterns of B lymphocyte infiltrates were found in the intestinal tissue: one was characterised by a strong to moderate stromal localisation of small IgM^+^/CD79^+^/CD20^-^/CD21^-^/CD23^-^/CD5^± ^IPCs (42.7% of cases); in the other (57.3%) no such small IPCs were detected in stromal or epithelial tissues. *IPCs *were significantly less frequent in the patients with Crohn's disease than in those with ulcerative colitis (p = 0.004).

**Conclusion:**

Our findings suggest that different immunopathogenetic pathways underlie chronic intestinal inflammation with different clinical expressions. The presence of small B lymphocytes resembling B-1 cells also seemed to be negatively associated with Crohn's disease. It can therefore be inferred that the gut contains an alternative population of B cells that have a regulatory function.

## Background

Crohn's disease (CD) and ulcerative colitis (UC) are idiopathic inflammatory bowel disorders [1] attributable to an abnormal immune response to bacterial antigens. Deficiencies in anti-inflammatory and immunosuppressive mechanisms are important to the development of the disease, but the basic pathogenetic mechanisms are still largely unknown [2].

Recently collated evidence supports the view that IBD consists of disorders with distinct genetic, microbial and environmental determinants that cluster into an UC or CD phenotype [3]. IBD is polygenic, and experimental data suggest that a number of not mutually exclusive pathways may contribute to the inflammatory cascades. CD has been attributed to the mediation of Th1, whereas UC shows a modified Th2 cytokine response [4].

Recent findings suggest that tissue injury in IBD is mediated by novel effector pathways, the most prominent of which is the interleukin-23/Th17 axis [5].

In both UC and CD, leukocyte recruitment is increased and this provides a potential target for therapeutic inhibition [3]. Effective defence against enteric pathogens requires leukocytes to be appropriately recruited and positioned in the gut to form an effective mucosal immune system.

The majority of in vivo studies of mouse intestinal B cells have shown that immunoglobulin-producing cells (IPCs) participate in the intestinal immune system by producing physiological intraluminal IgA and natural antibodies [6]. Pathologically atypical anti-neutrophil antibodies (xANCAs) may also be detected during the course of IBD [7].

Although > 80% of the B cells in the murine model are found in gut lymphoid tissue, it is actually unknown whether these derive from activated or recirculating B cells, or if they include populations of naïve B cells residing in the periphery [8].

However, it is known that the mammalian immune system contains a B-1 cell subset strategically positioned in the peritoneal and pleural cavities. These cells might migrate from the peritoneal cavity to a distant inflammatory lesion. Moreover they do not circulate through the lymph nodes, but migrate directly to the site of effector action.

These B-cells play a role in defending against infection during the period between activation of phagocytic cells (innate immunity) and T and B cells (adaptive immunity), and they also demonstrate the "promiscuous" expression of both myeloid and lymphoid characteristics [9]. In addition, B1 cells produce low-affinity antibodies, called natural antibodies, with limited diversity in the absence of infection.

The aim of this study was to evaluate the morphology, phenotype and tissue distribution, of IPCs in a substantial number of IBD patients in order to gain further insight into B cell pathobiology.

## Methods

### Patients

Small intestinal, colonic and rectal tissue samples were obtained from 96 patients undergoing complete colonoscopy at Fatebenefratelli Hospital in Milan, Italy. Biopsy specimens were taken from the inflamed mucosa and for each biopsy three sections were analysed. Informed consent was obtained from all of the patients before the procedure. The diagnosis of each case was confirmed using standard endoscopic and histological criteria (additional hematoxylin and eosin staining of each sample), and clinical data were obtained from clinical records. Sulphasalazine was used as a maintenance of treatment in the majority of the cases. If disease reactivation occurred, corticosteroids i.v. and infliximab were used. Control specimens were taken from ten patients *(30 sections) *with normal endoscopic findings and no macroscopic evidence of inflammatory or neoplastic disease. The biopsy sites were selected to obtain information from all parts of the intestinal tract.

### Immunofluorescence method

After deparaffinisation (72°C) and pre-treatment to enhance antigenicity (95°C × 36 min), we used indirect immunofluorescence (IFI) to detect the tissue expression distribution of immunoglobulins in intestinal sections (3 μm). The sections were incubated for 30 minutes with horse serum at room temperature in order to prevent non-specific binding. After removing the blocking solution, the sections were incubated with a polyclonal rabbit anti-human Ig (Santa Cruz Biotechnology, Santa Cruz, California, USA) or a goat anti-human IgM (μ chain specific, Vector Laboratories, Burlingame, California, USA). While being protected from direct light exposure at 37°C for one hour, the samples were washed four times for five minutes in PBS High Salt (NaCL 4 M and PO_4 _Buffer) and mounted. Images were taken from four randomly chosen sites on each sample (Euroimmun, Lubeck, Germany) at magnifications of 40× and 20×.

### Immunoperoxidase method

Murine monoclonal antibodies (mAbs) CD79, CD20, CD21, CD23 CD5, λ and κ chains were purchased from Medical Systems (Tucson, Arizona, USA) and used on formalin-fixed, paraffin-embedded tissue employing a Ventana automated slide stainer (Medical Systems). Biotinylated secondary antibodies were then added, followed by streptavidin-horseradish peroxidase conjugate. The anti-CD and anti λ and κ chains were optimally diluted for use with Ventana detection kits and automated slide stainers. Each stage of the staining protocol included incubation for a precise period of time at a specific temperature. At the end of each incubation stage, the sections were rinsed by the Ventana automated slide stainer to block the reaction and to remove any unbound material that might hinder desired reactions at subsequent stages. To minimise evaporation of the aqueous reagents from the specimen-containing slide, a coverslip solution was applied inside the slide stainer. The complex was then visualised using a hydrogen peroxide substrate and 3, 3'-diaminobenzidine tetrahydrochloride (DAB) chromogen.

### Clinical score

All patients in the study had an established diagnosis of CD or UC based on standard criteria (i.e. endoscopy, histology, barium contrast enema or other recognised criteria). Disease activity was defined using the Crohn's Disease Activity Index (CDAI) score of biological markers of inflammation or, in the case of UC [10-12], with the Disease Activity Index (DAI) validated by St Mark's Hospital and Academic Institute, England.

### Statistical analysis

The continuous variables were recorded as mean values and standard deviations and compared using an unpaired Student's t-test. The categorical variables were recorded as absolute and relative frequencies and compared using the Chi-squared test. The data were analysed using the Statistical Package for Social Sciences (SPSS 13.0; SPSS Inc., Chicago, IL, USA). All of the tests were two-sided and p values of < 0.05 were considered statistically significant.

## Results

### Study population

The study population comprised 96 patients with UC or CD, and consisted of more males than females. Their demographic and clinical characteristics are shown in Table 1.

**Table 1:** Characteristics of the patients included in the study

Patients	Ulcerative Colitis 64	Crohn's disease 32
**Males/females**	32/32	21/11
**Mean age at diagnosis, years**	44.2 ± 14.2	47.2 ± 16.6
**No therapy at diagnosis**	18 (28.1%)	16 (50.0%)
**Surgical therapy**	3 (4.7%)	10 (32.3%)
**Disease severity**		
• **Inactive** • **Mild** • **Moderate** • **Severe**	27 (42.2%) 19 (29.7%) 14 (21.9%) 4 (6.3%)	11 (34.4%) 15 (46.9%) 2 (6.3%) 4 (12.5%)

### IPC morphology and localisation can discriminate two types of cellular homing

**Figure 1:**
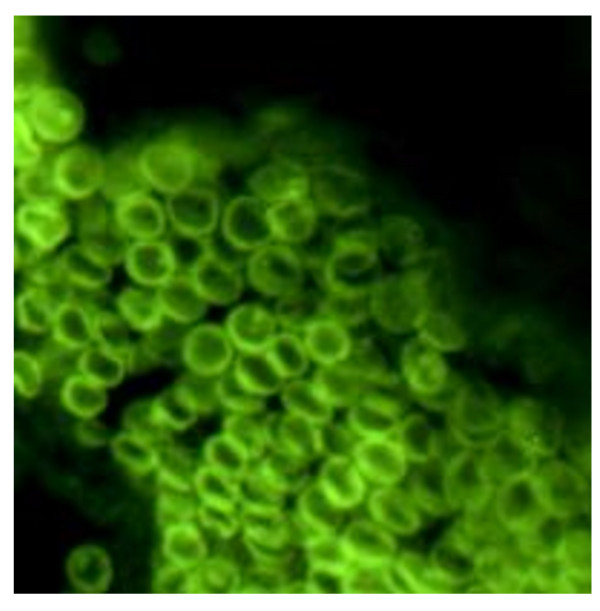
**Morphologic aspect of IPC in IFI method (Magnification 40×)**. These cells had a low cytoplasmic/nuclear ratio unlike conventional plasma cells. There is a fluorescent cut into the nucleus.

**Figure 2:**
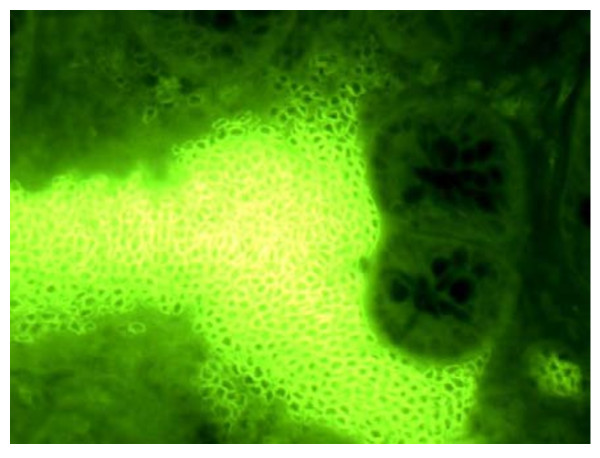
**Stromal and infraglandular distribution of monomorphic IPC (Magnification 40×)**. On using fluorescent antibodies anti chain μ, numerous small IPCs distributed within the glands, and characterised by a large central nucleus, were observed in the sections of inflamed mucosa.

**Figure 3:**
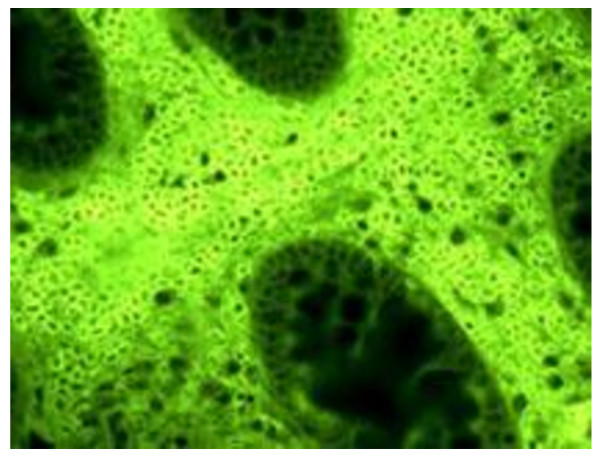
**Massive IPC Invasion seen on the intestinal tissue (Magnification 20×)**. In some biopsy specimens B-1 like cells were so numerous that they occupied all the periglandular space.

**Figure 4:**
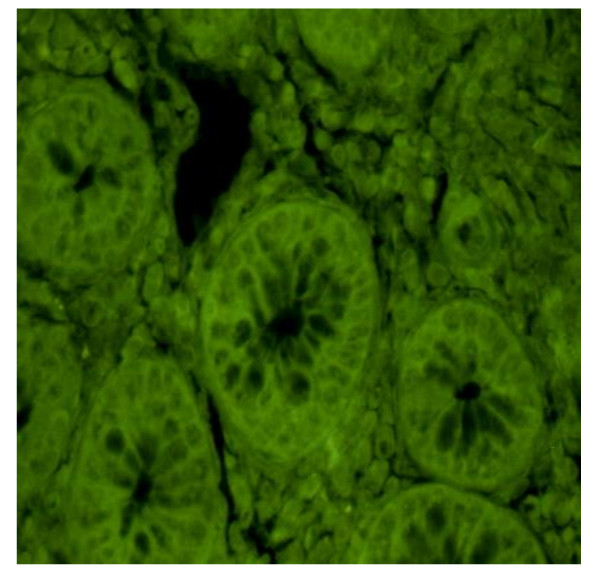
**Negative sample (Magnification 40×)**. In other sections and biopsies of inflamed mucosa taken from the same patient it is not possible to find the IPC on immunofluorescence.

Analysis of the morphology and distribution of IPCs in the biopsy specimens of inflamed tissue identified two subgroups of patients. 41 patients showed numerous small subepithelial IPCs distributed within the glands of the analysed specimens, which were characterised by a large central nucleus (Figure 1). These IPCs were surface IgM positive and monomorphic, and they often massively infiltrated inflamed tissues (Figures 2 and 3). Morphologically, they had a low cytoplasmic/nuclear ratio, unlike conventional plasma cells (Figure 4).

These were also present as discrete foci on ten normal small intestinal, colonic and rectal biopsy specimens., and seemingly contributed to the physiological intestinal cell population.

**Figure 5:**
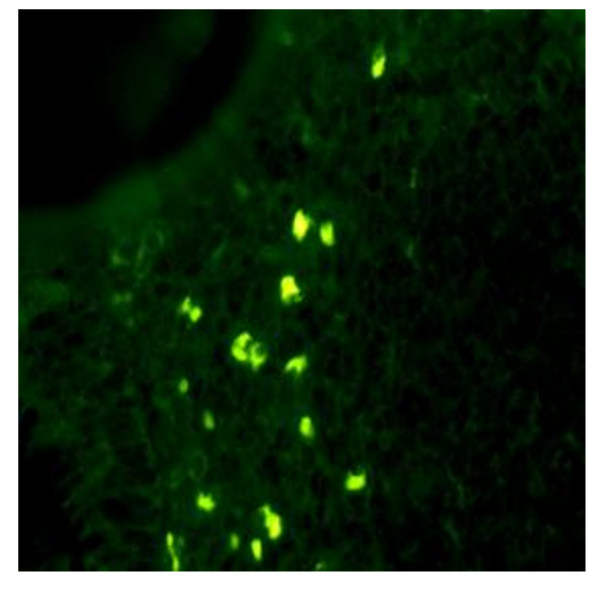
**Typical plasma cells with a "flame of the citoplasma" aspect/appearance (Magnification 40×)**. Characteristically this IPC had a sporadic distribution.

**Figure 6:**
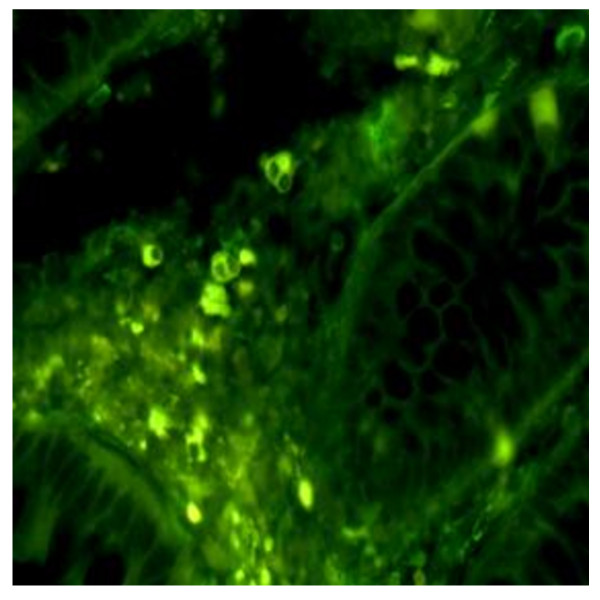
**IPC cells with irregular nuclei (Magnification 40×)**. These cells had a periglandular distribution and were characterized by positivity of the chain μ on imnunofluorescence even if the irregular aspect of the nucleous.

The remaining 55 patients had no such small IPCs across a number of different bowel tissue sections. The typical pattern in these cases was either a complete absence of IPCs (Figure 5) or evidence of a few typical plasma cells or IPCs with irregular nuclei (Figure 6). Characteristically, both types of IPC in this group were sporadically distributed and did not contribute to massive tissue infiltrates.

B cell surface phenotype analysis of inflammatory infiltrates identified a distinct CD79^+^/CD20^-^/CD21^-^/CD23^-^/CD5^± ^ICP subset.

B cell-specific surface molecules were examined in 20 representative samples taken from patients showing a large infiltrate of small IPCs with a low cytoplasmic/nuclear ratio. All of these samples originated from patients affected by UC.

All of the samples were CD79+ and κ and λ chain-positive. This finding was confirmed by IFI, thus supporting the fact that surface Ig expression can be used as a marker to identify B cells [13].

All of the samples were negative for three other markers specific to the B-2 cell subset: CD20 (a mature B cell-specific molecule), CD23 and CD21. To test whether the cells belonged to the B-1 subset, we analysed the surface expression of CD5, which was positive in 17 of the 20 samples. The CD5-negative samples were strongly positive for Ig and the symptoms of these patients were different from those observed in the CD5-positive group, particularly in terms of neurological manifestations. One of these patients had type one spino-cerebral ataxia and two female patients had peripheral neuropathy responsive to corticosteroid treatment. In one of these two cases this was associated with pluris nodular goiter micro and macro follicular.

The lack of CD79^+^/CD20^-^/CD21^-^/CD23^-^/CD5^± ^IPCs within the intestinal specimens was statistically associated with a diagnosis of CD (Table 2). The presence of B-1 like cells had a negative predictive value (NPV) of 82.9% for CD, with a sensitivity of 0.781 and a specificity of 0.531 (Table 2).

**Table 2:** Distribution of B cells by diagnosis

B-1-like cells	Ulcerative colitis (n = 64)	Crohn's disease (n = 32)	p	Sensitivity	Specificity	PPV	NPV
Highly represented	34 (53.1%)	7 (21.9%)	0.004	0.781	0.531	0.455	0.829
					
Absent	30 (46.9%)	25 (78.1%)					

Treatment did not influence CD79^+^/CD20^-^/CD21^-^/CD23^-^/CD5^± ^IPC expression (Table 3) as IPCs were observed with similar frequency in treated and untreated patients, and there was no significant difference between the IBD subgroups (Table 3).

**Table 3:** Distribution of B cells by diagnosis and treatment

B-1 cells highly represented	Therapy	No therapy	p
***All patients***	29/62 (46.8%)	12/34 (35.3%)	0.3
***UC***	4/16 (25.0%)	4/12 (18.8%)	0.7
***CD***	26/46 (56.5%)	8/18 (44.4%)	0.4

Only one of the 41 patients in the CD79^+^/CD20^-^/CD21^-^/CD23^-^/CD5^± ^IPC positive group had previously undergone surgical intervention for his/her disease during its clinical history compared to 13 out of 55 in the CD79^+^/CD20^-^/CD21^-^/CD23^-^/CD5^± ^IPC negative group. No significant statistical difference was found between these two groups in terms of anti-inflammatory and immunosuppressive drug use.

## Discussion

In this study, we identified an IgM^+ ^B cell subset in human intestinal tissue that is characterised by a CD79^+^/CD20^-^/CD21^-^/CD23^-^/CD5^± ^surface phenotype, which is atypical of B2 cells. CD5 (a marker that was initially used to distinguish B-1 from B-2 cells) [14] was often, but not always, co-expressed.

It is well-known that humans have one class of CD5^+ ^B cells that appear to share the phenotype properties of murine B-1a cells [15]. It has been reported that, like murine B-1a cells, CD5^+ ^human peripheral blood B cells produce polyspecific autoreactive antibodies [16,17]. It has been shown that CD5 is expressed on mouse B-1a but not B-1b cells, and its expression is largely extinguished during B-1a differentiation into antibody-secreting cells [18]. It has also been demonstrated that IL-9 restores a B-1 population in Xid mice, but exclusively with the B-1b surface phenotype; there, however, the *Xid*-B-1b cells failed to restore the classical functions of B-1a cells, thus indicating that this *Xid *sister population is functionally distinct from the B-1a cell subset [19].

Abrahão *et al*. identified B-1 cells using a colloidal gold immunocytochemical assay and found that mouse B-1a or B-1b cells have a single morphology that is distinct from that of B-2 cells. Mouse B-1 B cells are strategically positioned in the peritoneal and pleural cavities [20], but there is currently no marker or combination of markers that is expressed uniquely on all B-1 cells [18].

Shimomura *et al*. [21] described a previously unidentified subset of mouse IgM^+ ^B cells that present with an AA4.1^-^CD21^-^CD23^- ^major histocompatibility complex class II *(bright) *surface phenotype. These reside within the normal mucosa of the large intestine and expand in response to inflammation. This subset did not express B-1 cell markers (CD43, CD11b and CD5) but it proliferated *in vitro *in response to B cell receptor ligation and lipopolysaccharide stimulation. These cells appeared to exist in a unique pre-activated state and may originate from AA4.1^+ ^transitional B cells in the steady state. These B cells may be significantly increased in the inflamed intestine after recruitment from the recirculating naïve B cell pool.

In our study, we observed that the normal mucosa of the human intestine contains a CD79^+^/CD20^-^/CD21^-^/CD23^-^/CD5^± ^subset of IPCs. In 42.7% of the BD patients included in this study we observed a relevant, and sometimes massive, increase in these cells in the biopsies of inflamed intestinal tissue. Their absence, in turn, was associated with CD but unrelated to disease severity or treatment. Moreover, only one of the 41 patients in the CD79^+^/CD20^-^/CD21^-^/CD23^-^/CD5^□ ^IPC positive group had undergone surgical intervention for his/her disease during its clinical history, compared to 13 out of 55 in the CD79^+^/CD20^-^/CD21^-^/CD23^-^/CD5^□ ^IPC negative group. Thus, as shown above, B-1 cells might be active in intestinal inflammation, but their massive recruitment seems to be less consequential than their absence. IL-10 production is a well-known feature of the peritoneal CD5+ B1a subset, and it is interesting that a number of studies have shown that the phenotype of IL-10-producing Breg cells (CD11b^- ^CD5^- ^IgD^+^) is similar to that of B-2 (conventional B cells) but not to B-1a cells (CD11b^low ^CD5^+ ^IgD^-^) [22]. Experiments using co-cultures of B-1 cells and macrophages from Xid mice have demonstrated that B-1 cells down-regulate macrophage activity by means of IL-10 secretion [23], which illustrates their involvement in immune regulation.

In the subset of seemingly-negative B-1 cells, CD patients showed a periglandial infiltration compared with those patients with UC [Figure 6]. Our partial results demonstrate the presence of almost three different pathogenetic patterns of IPC found during the IBD evolution. The diagnosis of the patients analysed was related not only to the presence or absence of ICPs, but also to their distribution and morphology.

Moreover, because there were extra-intestinal manifestations in three patients with B-1 like cells and a CD5- phenotype, we could ask whether systemic mechanisms are involved in this subgroup of patients.

## Conclusion

The immune system has developed many different mechanisms to regulate immune responses, and B cells also seem to play a pathogenic role in inflammation. Autoimmune diseases are often characterised by the co-existence of clinical features compatible with chronic inflammatory conditions, with a variable prevalence of different components. The finding that IPCs derived from innate immunity B cells are present in inflamed tissues in close contact with several environmental stimuli strengthens the hypothesis that microbes can induce autoimmune responses [24]. However further studies are needed to clarify the role of cells linking innate to adaptive immunity, including their homing potential, to different tissues.

## Abbreviations

IBD: inflammatory bowel disease; IPC: immunoglobulin-producing cell; IFI: indirect immuno-fluorescence; xANCA: atypical cytoplasmic and perinuclear anti-neutrophil cytoplasmic antibody; TLR: Toll-like receptor; NOD: nucleotide-binding oligomerisation domain; UC: ulcerative colitis; CD: Crohn's disease; TNF: tumour necrosis factor; IL: interleukin; mAb: murine monoclonal antibody: DAB: 3,3'-diaminobenzidine tetrahydrochloride; PPV: positive predictive value; NPV: negative predictive value.

## Competing interests

The authors declare that they have no competing interests.

## Authors' contributions

CD and SG carried out the Immunofluorescence and Immunoperoxidase method and drafted the manuscript. OS, AC, SS and SB participated in the coordination of the study. CD, FA, PSP participated in the design and coordination of the study and helped to draft the manuscript. MA participated in the study and performed the statistical analysis. SB participated in the design of the study and performed the statistical analysis. PLA conceived of the study, and participated in its design and coordination. All authors read and approved the final manuscript.
